# The CCHCR1–UBAP2L Interaction Promotes UBAP2L Release from P-Bodies for Stress Granule Assembly

**DOI:** 10.3390/ijms27167451

**Published:** 2026-08-20

**Authors:** Zhaohui Ye, Mingze Xu, Chun Lin, Stephen Cho Wing Sze, Chunman Li

**Affiliations:** 1 Shantou University Medical College, Shantou 515041, China; 23zhye5@stu.edu.cn; 2 Department of Anatomy, Shantou University Medical College, Shantou 515041, China; 25mzxu@stu.edu.cn (M.X.); seplin@stu.edu.cn (C.L.); 3 Research Unit, Bio Technology and Natural Products for Health Development Limited, Hong Kong Baptist University Startup, Hong Kong, China; stephensze@gmail.com

**Keywords:** RNA granule dynamics, CCHCR1, UBAP2L, liquid–liquid phase separation

## Abstract

Ribonucleoprotein granules such as processing bodies (P-bodies) and stress granules (SGs) are membrane-less organelles that regulate mRNA metabolism through liquid–liquid phase separation. UBAP2L drives SG assembly and can bridge P-bodies with SGs, yet how it is mobilized between these compartments remains unclear. Here, using co-immunoprecipitation, GST pull-down, CRISPR-Cas9-mediated knockout, and immunofluorescence microscopy, we demonstrate that CCHCR1 directly binds UBAP2L and that this interaction is dynamically regulated by stress intensity. Under mild oxidative stress, CCHCR1 retains UBAP2L in P-bodies; as stress intensifies, this interaction weakens, permitting UBAP2L release for SG assembly. CCHCR1 deficiency aberrantly traps UBAP2L in P-bodies via enhanced DDX6 association, resulting in defective SG assembly, delayed maturation, and increased P-body–SG fusion. These findings establish CCHCR1 as a stress-responsive switch that controls UBAP2L partitioning between P-bodies and stress granules, thereby controlling the threshold and kinetics of SG biogenesis.

## 1. Introduction

Ribonucleoprotein granules form in the cytoplasm through phase separation, creating membrane-free compartments that organize mRNA metabolism [1,2,3,4]. Processing bodies house translationally silent transcripts and decay machinery under basal conditions, while stress granules assemble transiently upon environmental stress to arrest translation and store mRNPs [5,6]. The dynamic interplay between processing bodies and stress granules has long been recognized, yet the mechanisms that govern their functional coordination remain elusive [3,7,8]. Recent studies have revealed that the DEAD-box helicase DDX6 localizes to P-bodies and restricts SG assembly by binding granule-associated RNAs and hydrolyzing ATP to release proteins and RNAs, thereby preventing excessive granule accumulation [9,10].

Other P-body components, including the decapping machinery DCP1/DCP2 and the 5′-3′ exonuclease XRN1, have also been observed in stress granules under certain conditions [3,11]. This functional duality suggests that the distribution of shared components is actively regulated. UBAP2L, initially annotated as a ubiquitin-associated protein based on sequence prediction, drives stress granule assembly through G3BP1 and G3BP2 [2,12,13]. It is diffuse in the cytoplasm under basal conditions, accumulates in P-bodies or stress granules depending on stress intensity, and its overexpression can bridge the two compartments into hybrid condensates [14]. Post-translational modifications of UBAP2L further modulate SG behavior—arginine methylation by PRMT1 suppresses assembly and promotes disassembly, while O-GlcNAcylation enhances SG formation and has been linked to sunitinib resistance in renal cell carcinoma [15,16]. Together, these findings establish UBAP2L as a key node in the regulatory network that mediates P-body–stress granule communication.

CCHCR1 was first identified as a psoriasis susceptibility gene within the PSORS1 locus on chromosome 6p21.3 [17]. Early genetic studies linked CCHCR1 variants to disease risk, yet the molecular basis of this association remained obscure [18,19,20,21,22]. Proteomic screening identified several CCHCR1-interacting proteins, predominantly centrosomal and P-body components [23,24]. Subsequent studies confirmed that CCHCR1 localizes to both centrosomes and P-bodies [25,26]. At centrosomes, CCHCR1 interacts with astrin to recruit CEP72 and downstream MCPH proteins, thereby promoting centriole duplication and maintaining microtubule organization [24]. In P-bodies, CCHCR1 binds EDC4 through its N-terminal coiled-coil domain [23]. Although our previous work showed that CCHCR1 bridges P-bodies to the centrosome by binding EDC4 at the N-terminus and OFD1/PCM1 at the C-terminus, and that this tethering is essential for primary cilia formation, the function of CCHCR1 within P-bodies beyond this structural anchoring remains unclear [26].

Here, we report that CCHCR1 binds UBAP2L and controls its release from P-bodies for stress granule assembly. This interaction is direct, stress-responsive, and weakens UBAP2L association with DDX6. CCHCR1 deficiency enhances DDX6 binding to UBAP2L and traps UBAP2L in P-bodies, resulting in defective stress granule formation and stability. These findings establish CCHCR1 as a switch that mobilizes P-body factors for stress granule biogenesis.

## 2. Results

### 2.1. CCHCR1 Interacts with UBAP2L and This Interaction Is Regulated by Stress Intensity

**Figure 1:**
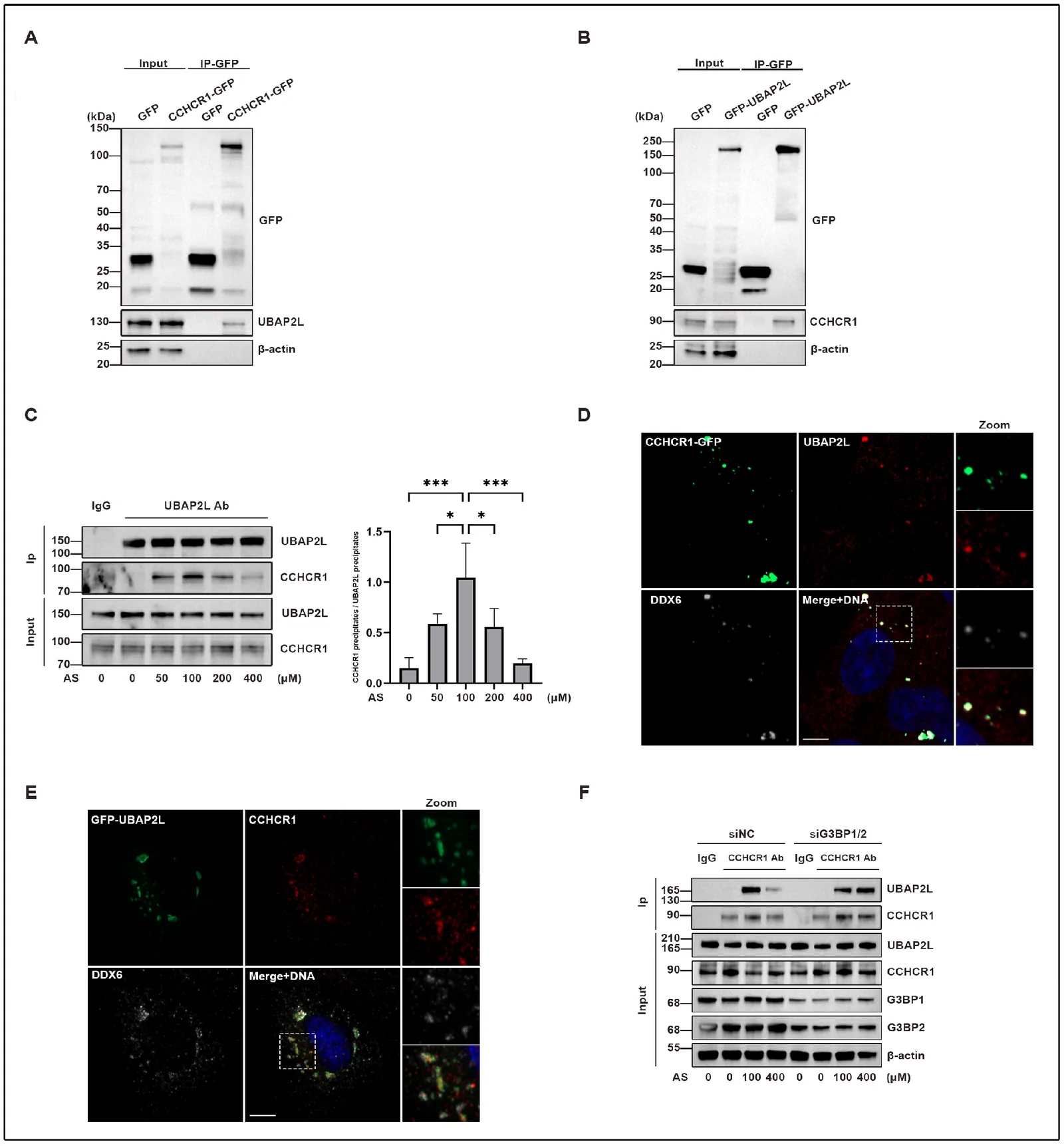
CCHCR1 interacts with UBAP2L in a stress-intensity-dependent manner. (**A**) Co-immunoprecipitation (Co-IP) of CCHCR1-GFP with endogenous UBAP2L. Lysates of HEK293 cells expressing CCHCR1-GFP were immunoprecipitated with anti-GFP antibody, using mouse IgG as a negative control. The lysates and precipitates were subjected to immunoblotting with the indicated antibodies. (**B**) Reciprocal Co-IP of GFP-UBAP2L with endogenous CCHCR1. Lysates of HEK293 cells expressing GFP-UBAP2L were immunoprecipitated with anti-GFP antibody and analyzed by immunoblotting. (**C**) Endogenous Co-IP of CCHCR1 and UBAP2L in HEK293 cells treated with the indicated concentrations of sodium arsenite for 50 min. Cell lysates were immunoprecipitated with anti-CCHCR1 antibody, and the precipitates were probed for UBAP2L and CCHCR1. Quantitative analysis of CCHCR1 band intensity in precipitates normalized to the precipitated UBAP2L was performed using three biological replicates. Data represent mean ± SD. * *p* < 0.05, *** *p* < 0.001 (one-way ANOVA with Dunnett’s post hoc test). (**D**) Immunofluorescence images of HEK293 cells expressing CCHCR1-GFP (green) and stained with antibodies against UBAP2L (red) and DDX6 (gray); nuclei were labeled with DAPI (blue); the boxed region is magnified in the zoom panel; scale bar, 5 μm. (**E**) Immunofluorescence images of HEK293 cells expressing GFP-UBAP2L (green) and stained with antibodies against CCHCR1 (red) and DDX6 (gray); nuclei were labeled with DAPI (blue); the boxed region is magnified in the zoom panel; scale bar, 5 μm. (**F**) Co-IP of endogenous CCHCR1 with UBAP2L in G3BP1/2 double-knockdown cells across arsenite concentrations. G3BP1/2 double-knockdown HEK293 cells were treated with the indicated concentrations of sodium arsenite for 50 min. Cell lysates were immunoprecipitated with anti-CCHCR1 antibody, and precipitates were subjected to immunoblotting for the indicated proteins. Input lysates are shown in the lower panels.

In our previous BioID screen using CCHCR1 as bait, we identified a series of candidate interacting proteins, among which UBAP2L was of particular interest due to its established role in stress granule assembly [24,26]. To validate this interaction, co-immunoprecipitation (co-IP) assays were performed. We found that overexpressed CCHCR1-GFP efficiently co-precipitated endogenous UBAP2L (Figure 1A). In a reciprocal experiment, overexpressed GFP-UBAP2L was able to co-precipitate endogenous CCHCR1 (Figure 1B), confirming a specific interaction between CCHCR1 and UBAP2L.

**Figure A1:**
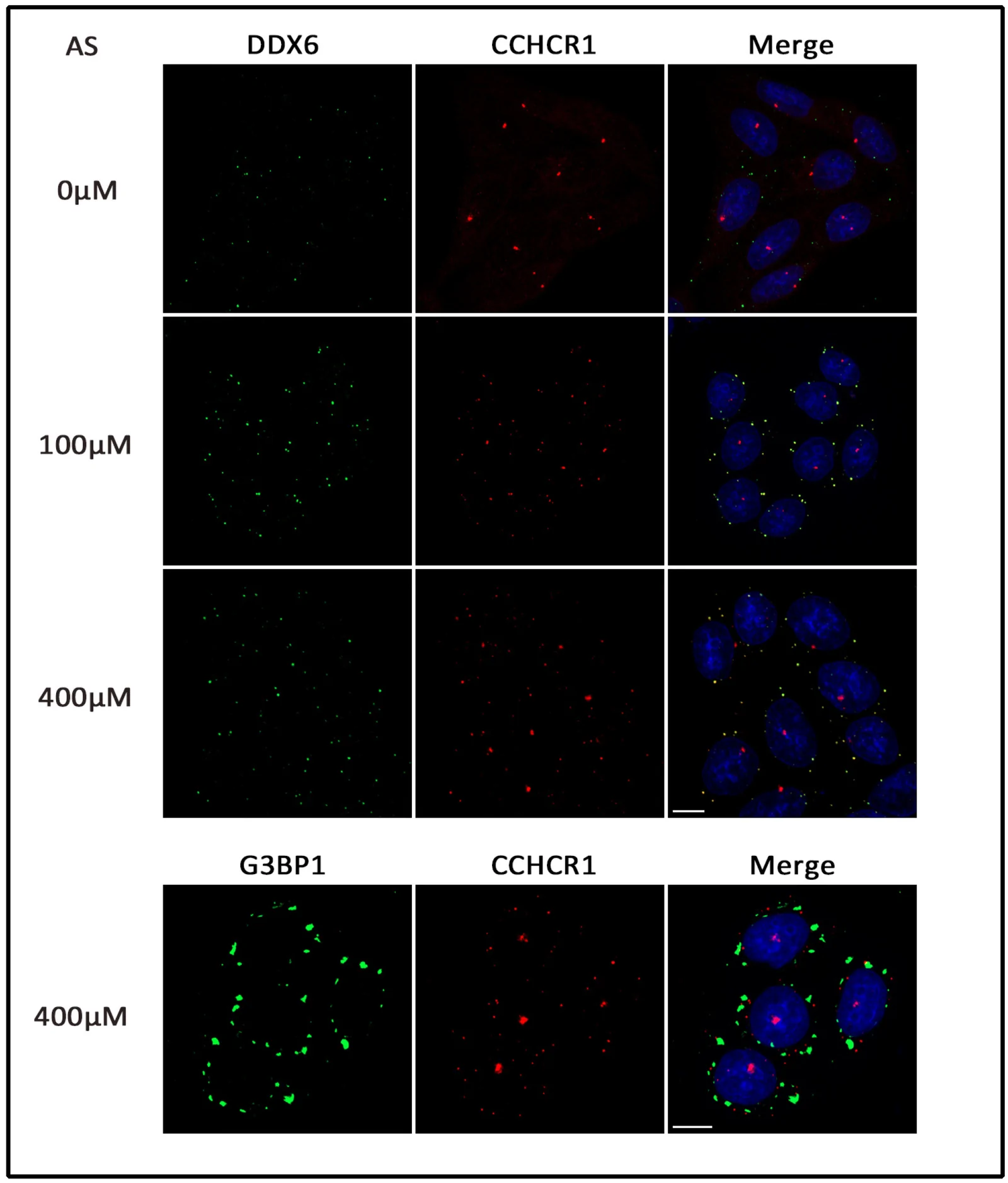
Endogenous CCHCR1 localizes to P-bodies under arsenite-induced oxidative stress. HEK293 cells were immunostained for endogenous CCHCR1 (red) and the P-body marker DDX6 (green) or SG marker G3BP1 (green), treated with the indicated arsenite for 50 min. Scale bars, 10 μm.

**Figure A2:**
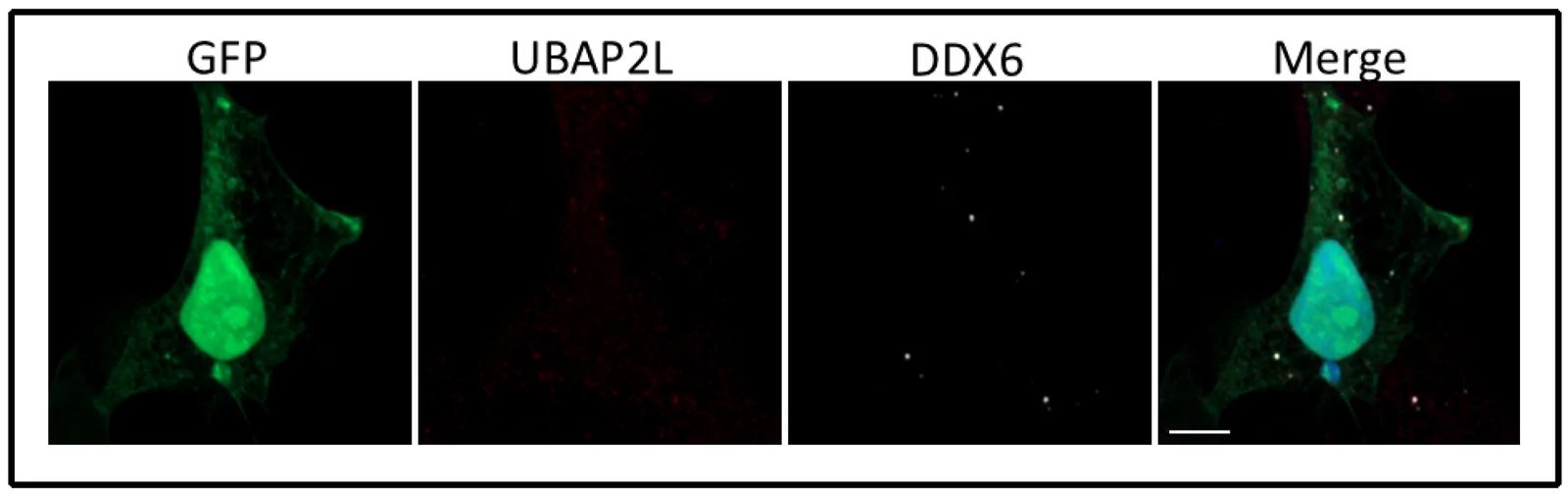
GFP alone does not recruit UBAP2L to P-bodies. HEK293 cells overexpressing GFP (green) alone were immunostained for endogenous UBAP2L (red) and the P-body marker DDX6 (gray). Note the predominantly diffuse cytoplasmic distribution of UBAP2L with only a weak P-body signal. Scale bar, 5 μm.

**Figure A3:**
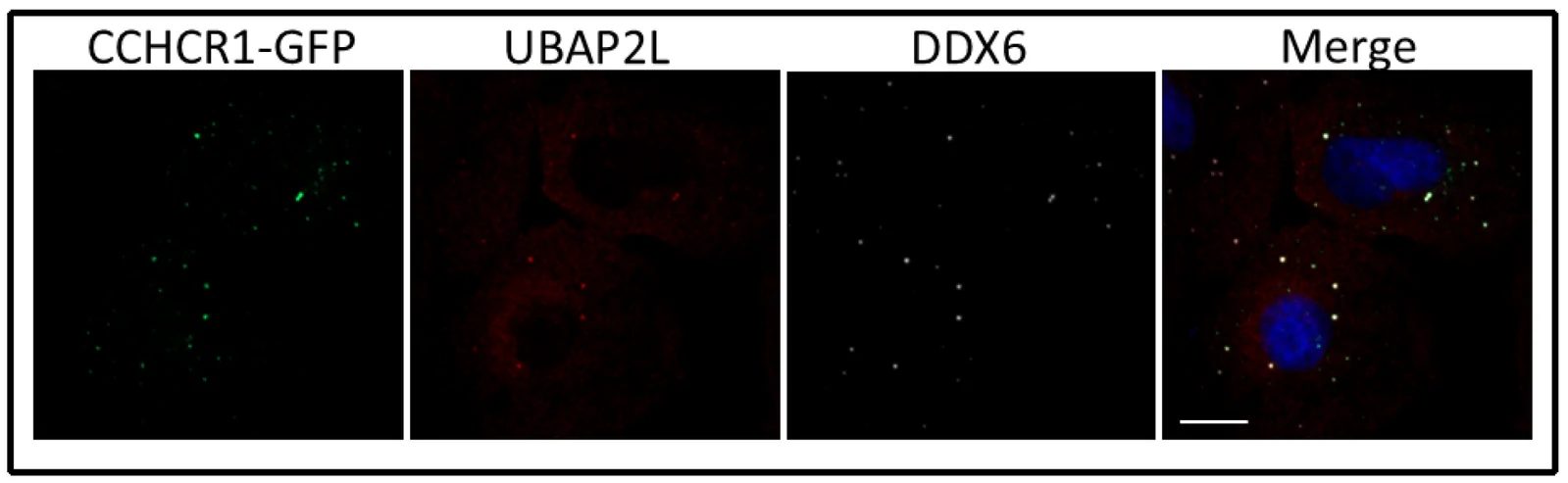
CCHCR1–UBAP2L co-localization at P-bodies is conserved in U2OS cells. U2OS cells transfected with CCHCR1-GFP (green) were stained for endogenous UBAP2L (red) and DDX6 (gray), scale bar, 10 μm.

However, under normal cell culture conditions, co-IP did not detect an interaction between endogenous CCHCR1 and UBAP2L. While UBAP2L has been reported to localize to P-bodies and stress granules under varying intensities of oxidative stress [14], the subcellular distribution of endogenous CCHCR1 is less clear. Previous studies have described CCHCR1 as a P-body and centrosome-localized protein [23,25,26]; however, we noticed that evidence for its P-body localization derives almost exclusively from overexpression systems. To examine the behavior of the endogenous protein, we assessed CCHCR1 localization across a range of arsenite concentrations. In unstressed HEK293 cells, endogenous CCHCR1 exhibited a diffuse cytoplasmic pattern with pronounced enrichment at centrosomes. Upon treatment with 100 µM arsenite for 50 min, CCHCR1 was recruited to P-bodies, as evidenced by co-localization with DDX6. In addition, even under severe stress (400 µM arsenite), CCHCR1 remained associated with P-bodies and did not co-localize with the stress granule marker G3BP1 (Figure A1). Therefore, endogenous co-IP was performed across a range of arsenite concentrations to examine whether P-body accumulation and SG formation affect the CCHCR1–UBAP2L interaction. Strikingly, the CCHCR1–UBAP2L association became detectable at 50 μM arsenite, peaked at 100 μM, and progressively declined at 200 and 400 μM (Figure 1C). This finding reveals a dynamic interaction that is induced during mild-to-moderate oxidative stress but progressively weakens as stress intensity rises. Immunofluorescence assays showed that overexpressed CCHCR1-GFP exhibited a punctate cytoplasmic distribution and co-localized with both the P-body marker DDX6 and UBAP2L (Figure 1D). In contrast, when GFP alone was overexpressed, UBAP2L was predominantly diffusely distributed in the cytoplasm, with only a weak signal detected in P-bodies (Figure A2). The CCHCR1–UBAP2L co-localization at P-bodies was also reproduced in U2OS cells (Figure A3). These results demonstrate that ectopic expression of CCHCR1 is sufficient to recruit UBAP2L to P-bodies.

Next, when GFP-UBAP2L was overexpressed, it formed large cytoplasmic granules that co-localized with both endogenous CCHCR1 and DDX6, suggesting that UBAP2L, when locally enriched, can also coalesce at CCHCR1-marked P-bodies (Figure 1E).

To test whether arsenite directly disrupts the CCHCR1–UBAP2L interaction, we examined G3BP1/2 double-knockdown cells. If arsenite chemically modified either protein to destabilize their binding, the interaction should weaken regardless of G3BP1/2 status. However, Co-IP revealed that CCHCR1–UBAP2L binding persists in G3BP1/2 DKO cells treated with 400 µM arsenite for 50 min (Figure 1F). This indicates that arsenite-induced modification alone is insufficient to dissociate CCHCR1–UBAP2L. Given that stress-induced UBAP2L demethylation increases its affinity for G3BP1 [15], the weakening of the CCHCR1–UBAP2L interaction observed under severe stress may reflect a G3BP1-dependent competitive binding of UBAP2L.

Taken together, these findings establish that CCHCR1 physically interacts with UBAP2L and recruits it to P-bodies, and that this interaction is dynamically regulated by stress intensity.

### 2.2. The N-Terminal Coiled-Coil Domain of CCHCR1 Binds and Targets UBAP2L to P-Bodies

**Figure 2:**
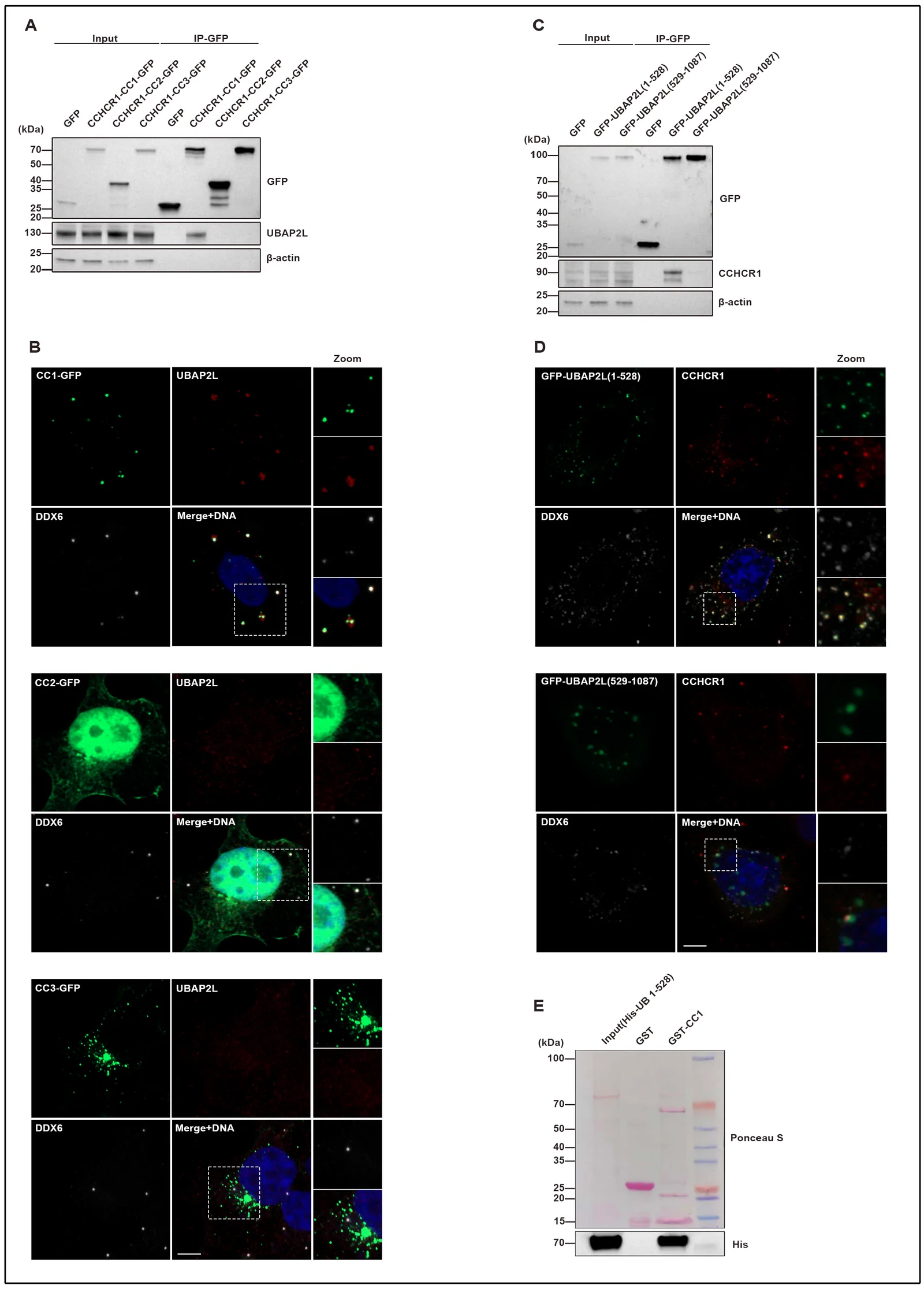
The N-terminal coiled-coil domain of CCHCR1 binds the N-terminal region of UBAP2L. (**A**) Co-IP of GFP-tagged CCHCR1 truncation mutants (CC1, CC2 and CC3) with endogenous UBAP2L. Lysates of HEK293 cells expressing the indicated constructs were immunoprecipitated with anti-GFP antibody, using mouse IgG as a negative control. The lysates and precipitates were subjected to immunoblotting for the indicated antibodies. (**B**) Immunofluorescence images of HEK293 cells expressing GFP-tagged CCHCR1 fragments (green), and stained with antibodies against UBAP2L (red) and DDX6 (gray); nuclei were labeled with DAPI (blue). The boxed region is magnified in the zoom panel; scale bar, 5 μm. (**C**) Co-IP of GFP-tagged UBAP2L truncation mutants (aa. 1–528 and aa. 529–1087) with endogenous CCHCR1. Lysates of HEK293 cells expressing the indicated constructs were immunoprecipitated with anti-GFP antibody. The lysates and precipitates were subjected to immunoblotting for the indicated antibodies. (**D**) Immunofluorescence images of HEK293 cells expressing GFP-UBAP2L (aa. 1–528 or 529–1087, green), and stained with antibodies against CCHCR1 (red) and DDX6 (gray); nuclei were labeled with DAPI (blue). The boxed region is magnified in the zoom panel scale bar, 5 μm. (**E**) GST pull-down of GST-CCHCR1-CC1 (GST-CC1) with His-UBAP2L (1–528) (His-UB 1–528). GST alone served as a negative control. Input and bound fractions were analyzed by immunoblotting with anti-His antibody (bottom panel) and Ponceau S staining (top panel).

Our previous study demonstrated that the three coiled-coil domains of CCHCR1 have distinct subcellular targeting properties: the N-terminal coiled coil (CC1) directs P-body localization, the C-terminal coiled coil (CC3) directs centrosome localization, and the middle coiled coil (CC2) shows no specific targeting [26]. To determine which region of CCHCR1 mediates the interaction with UBAP2L, we generated GFP-tagged truncation mutants and performed co-immunoprecipitation assays. The CC1-containing CCHCR1 fragment co-precipitated UBAP2L, whereas the CC3-containing fragment did not (Figure 2A). Consistently, immunofluorescence analysis showed that the CC1-containing fragment localized to P-bodies and recruited UBAP2L to these structures. In contrast, the CC3-containing fragment, although forming granular structures, did not co-localize with P-bodies and failed to recruit UBAP2L (Figure 2B). These results indicate that the CC1 domain of CCHCR1 is both necessary and sufficient for binding UBAP2L and targeting it to P-bodies. To map the corresponding region in UBAP2L, we constructed GFP-tagged truncation mutants based on the domain architecture of UBAP2L and previous reports. UBAP2L contains an N-terminal ubiquitin-associated (UBA) domain (aa 49–89), an Arg-Gly-Gly (RGG) motif (aa 131–190), three predicted RNA-binding regions (aa 239–257, 282–290, and 850–864), and a domain of unknown function (DUF, aa 495–526) [27,28]. We found that UBAP2L (aa 1–528), but not UBAP2L (aa 529–1087), co-precipitated CCHCR1 (Figure 2C). Immunofluorescence analysis confirmed that GFP-UBAP2L (aa 1–528) co-localized with endogenous CCHCR1 and the P-body marker DDX6 (Figure 2D). However, further subdivision of the UBAP2L (aa 1–528) region did not identify a more precise interaction motif, suggesting that the binding interface may require the overall conformation of this region rather than a discrete sequence. To determine whether the CCHCR1-UBAP2L interaction is direct, we performed GST pull-down assays using purified recombinant proteins. GST-CCHCR1-CC1, but not GST alone, efficiently pulled down His-tagged UBAP2L (aa 1–528) (Figure 2E). Taken together, these findings establish that CCHCR1 directly interacts with UBAP2L through the CC1 domain of CCHCR1 and the N-terminal region (aa 1–528) of UBAP2L.

### 2.3. CCHCR1 Deficiency Traps UBAP2L in P-Bodies and Impairs Stress Granule Assembly

To investigate the physiological role of the CCHCR1–UBAP2L interaction, we generated *CCHCR1*-knockout (KO) HEK293 cells using CRISPR-Cas9 and examined UBAP2L localization under varying arsenite concentrations in both wild-type and KO cells.

**Figure 3:**
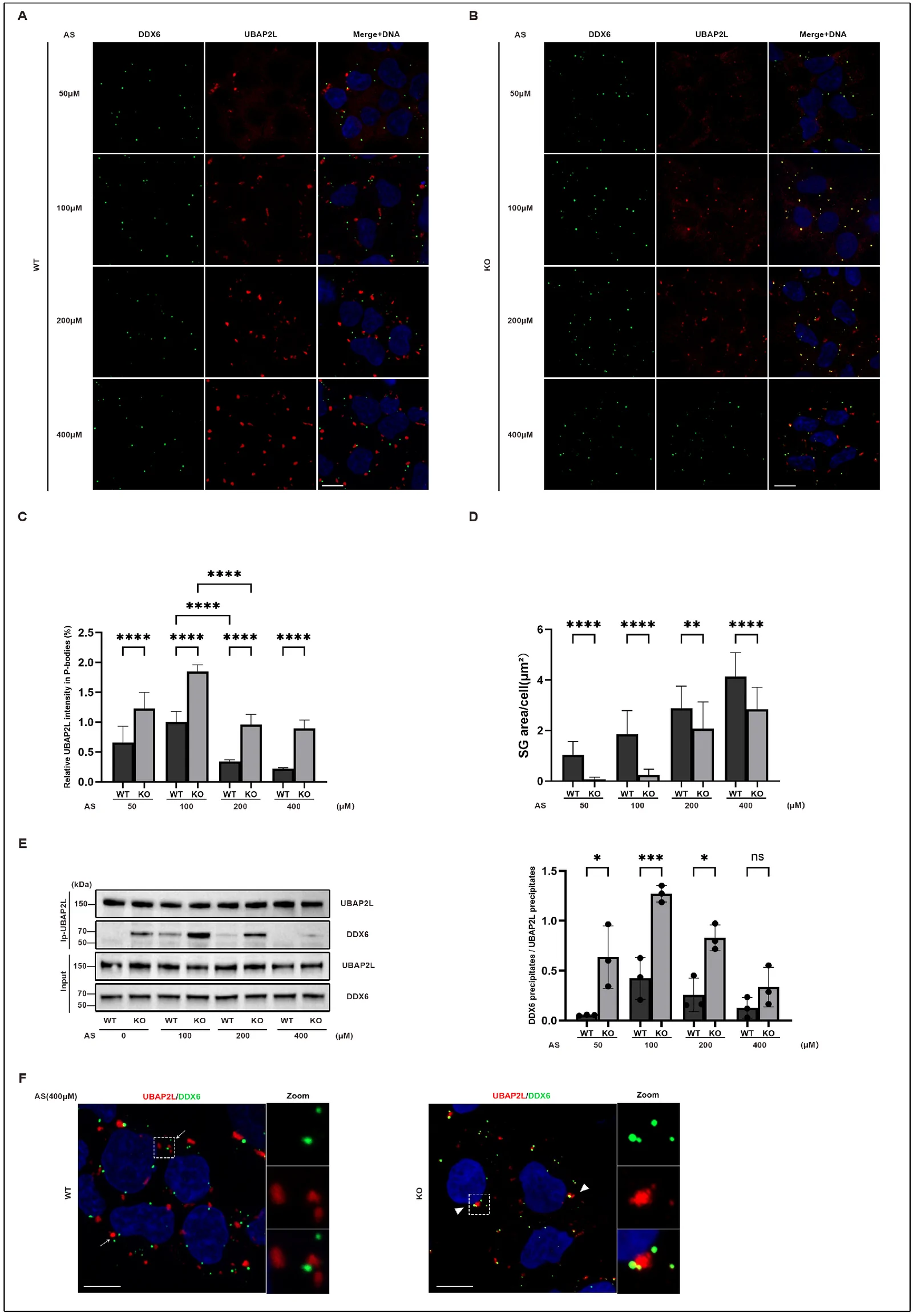
CCHCR1 deficiency traps UBAP2L in P-bodies and impairs stress granule dynamics. (**A**,**B**) Immunofluorescence images of wild-type (WT, **A**) and *CCHCR1*-KO (KO, **B**) HEK293 cells stained for DDX6 (green), UBAP2L (red), and DAPI (blue) at indicated arsenite concentrations. Scale bars, 10 μm. (**C**) Quantification of UBAP2L retention in P-bodies. UBAP2L mean fluorescence intensity in DDX6-positive P-bodies was measured in wild-type and *CCHCR1*-KO cells under indicated arsenite concentrations. *n* = 30 cells per condition from 3 independent experiments. Data represent mean ± SD. **** *p* < 0.0001 (one-way ANOVA with Dunnett’s post hoc test). (**D**) Quantification of average SG area per cell at indicated arsenite concentrations. At least 30 cells (*n* = 30) per condition were counted across three independent experiments. Data represent mean ± SD. ** *p* < 0.01, **** *p* < 0.0001 (one-way ANOVA with Dunnett’s post hoc test). (**E**) Co-immunoprecipitation of endogenous UBAP2L with DDX6 at the indicated arsenite concentrations in wild-type and *CCHCR1*-KO cells. Lysates of HEK293 cells were immunoprecipitated with anti-UBAP2L antibody. The lysates and precipitates were subjected to immunoblotting with the indicated antibodies. Quantitative analysis of DDX6 band intensity in precipitates normalized to precipitated UBAP2L was performed using three biological replicates. Data represent mean ± SD. * *p* < 0.05, *** *p* < 0.001, ns means *p* > 0.05 (one-way ANOVA with Dunnett’s post hoc test). (**F**) Immunofluorescence images showing the spatial relationship between SGs (G3BP1, green) and P-bodies (DDX6, red) at 400 μM arsenite for 50 min in wild-type (WT) and *CCHCR1*-KO (KO) HEK293 cells. Arrowheads indicate SGs adjacent to or partially overlapping with P-bodies in KO cells; arrows denote spatially segregated SGs and P-bodies in wild-type cells. Scale bars, 10 μm.

**Figure A4:**
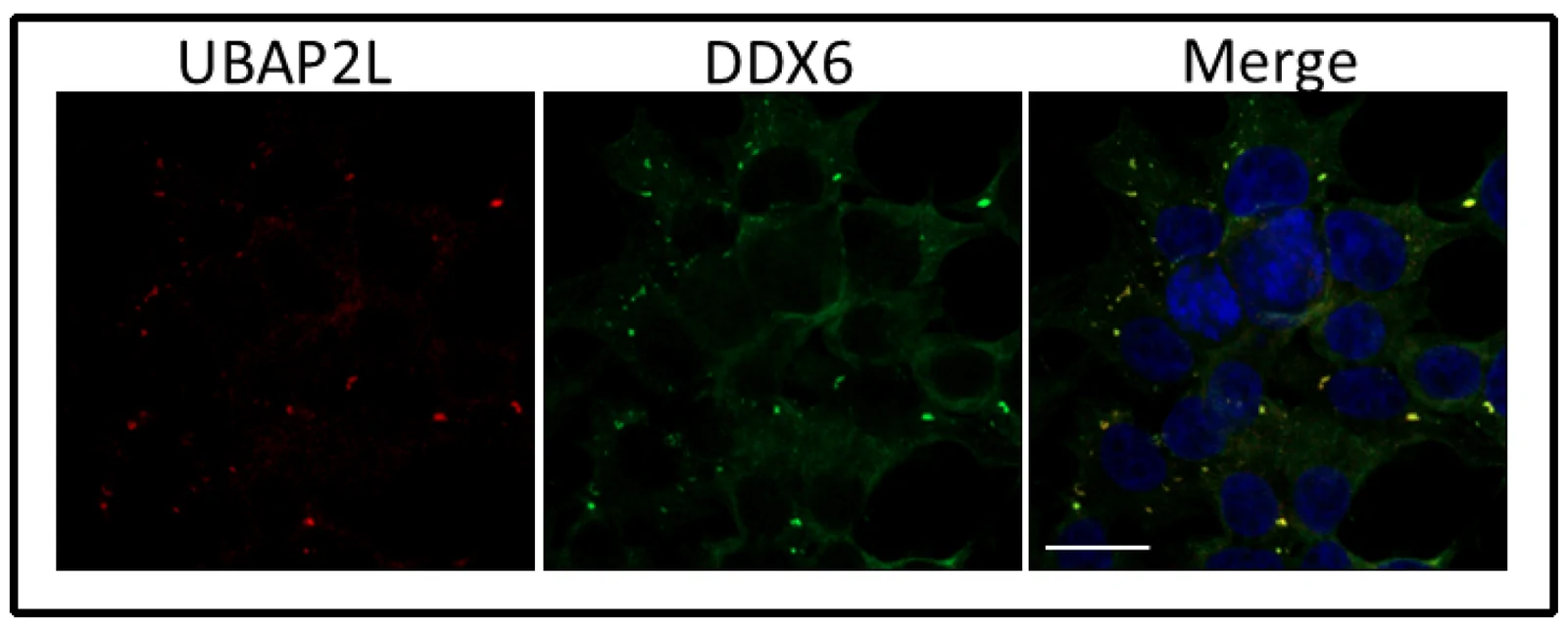
G3BP1 staining confirms UBAP2L condensates as stress granules. HEK293 cells were treated with 100 μM sodium arsenite for 50 min and co-stained for UBAP2L (red) and the stress granule marker G3BP1 (green). Scale bar, 20 μm.

**Figure A5:**
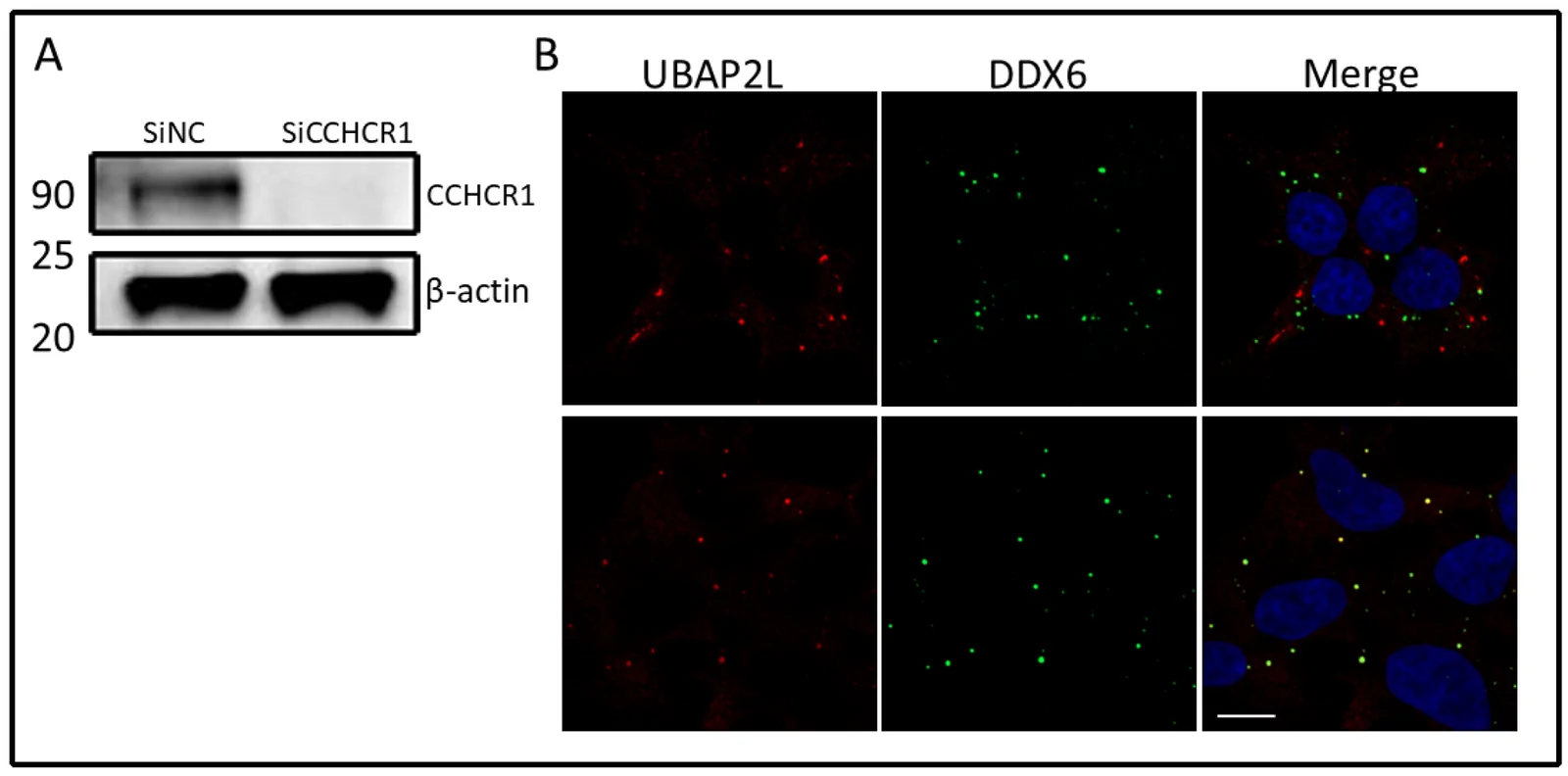
siRNA-mediated CCHCR1 knockdown phenocopies the knockout phenotype. (**A**) Western blot confirming *CCHCR1* knockdown efficiency. (**B**) Immunofluorescence showing UBAP2L (red) localization in control and *CCHCR1*-siRNA cells treated with 100 μM arsenite. Note enhanced P-body retention (DDX6, green) in knockdown cells. Scale bar, 10 μm.

At 50 μM arsenite, UBAP2L exhibited a heterogeneous distribution in wild-type cells. Some cells showed punctate signals overlapping with DDX6, indicating P-body entry, while a small subset displayed larger UBAP2L condensates that did not overlap with DDX6. At 100 μM, these larger aggregates became more prominent, whereas P-body-associated UBAP2L was barely detectable. Immunofluorescence staining with the SG marker G3BP1 confirmed that these condensates were stress granules (Figure A4). At 200 μM or higher, UBAP2L signals were exclusively localized to stress granules (Figure 3A). In contrast, *CCHCR1*-KO cells showed markedly different behavior. At 50 μM arsenite, UBAP2L was predominantly localized to DDX6-positive P-bodies. This accumulation became more pronounced at 100 μM, with almost no UBAP2L puncta detected outside P-bodies. At 200 μM, UBAP2L signals were partially retained in P-bodies and partially appeared in stress granules. Even at 400 μM, although most UBAP2L had translocated to stress granules, a residual signal remained in DDX6-positive P-bodies (Figure 3B). siRNA-mediated knockdown of CCHCR1 phenocopied the KO cells, confirming that the observed UBAP2L retention in P-bodies was specific to CCHCR1 loss (Figure A5). Quantification of UBAP2L mean fluorescence intensity within DDX6-positive P-bodies confirmed enhanced retention in *CCHCR1*-KO cells. At 100 μM arsenite, KO cells showed ~85% higher P-body UBAP2L intensity than wild-type cells. This elevation persisted at 200 μM but declined at 400 μM, whereas signals in wild-type cells were minimal at this concentration (Figure 3C). In addition, the average SG area per cell was reduced by ~28% and ~41% at 200 μM and 400 μM arsenite, respectively, in KO cells compared to wild-type, while SG number per cell remained comparable between genotypes (Figure 3D). These observations suggest that CCHCR1 deficiency not only prevents UBAP2L exit from P-bodies but also strengthens its association with P-bodies. To test this directly, we performed co-immunoprecipitation assays to compare endogenous UBAP2L–DDX6 association across arsenite concentrations. Consistent with the imaging data, UBAP2L co-precipitated significantly more DDX6 in CCHCR1-KO cells than in wild-type cells at all tested concentrations (0, 100, 200, and 400 μM). At 100 μM arsenite—the peak of the CCHCR1–UBAP2L interaction—the amount of DDX6 bound to UBAP2L in KO cells was more than three-fold higher than in wild-type cells (Figure 3E).

These findings demonstrate that CCHCR1 deficiency causes aberrant UBAP2L retention in P-bodies under low-to-moderate stress and impairs SG maturation even under severe stress conditions.

Notably, at 400 μM arsenite, although most UBAP2L had translocated to stress granules in both genotypes, *CCHCR1*-KO cells exhibited an increased number of SGs adjacent to or partially overlapping DDX6-positive P-bodies, compared to wild-type cells, where SGs and P-bodies remained largely spatially segregated (Figure 3F). This observation indicates that loss of CCHCR1 compromises the spatial separation between SGs and P-bodies even under severe stress conditions.

### 2.4. CCHCR1 Is Required for Efficient Stress Granule Assembly and Disassembly Dynamics

**Figure 4:**
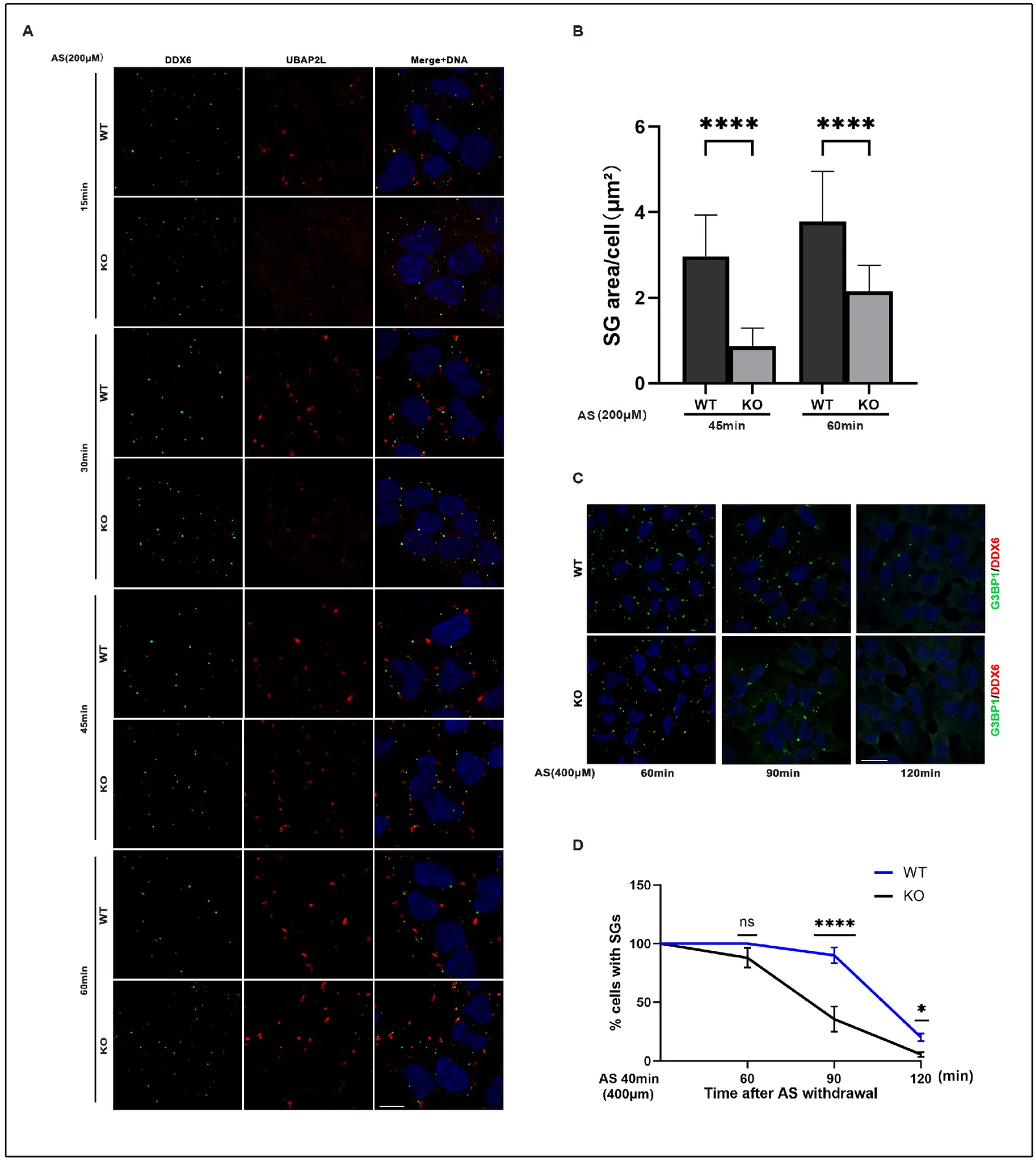
CCHCR1 is required for efficient stress granule assembly and disassembly dynamics. (**A**) Representative immunofluorescence images of wild-type (WT) and *CCHCR1*-KO (KO) HEK293 cells stained for UBAP2L (red) and DDX6 (green), at indicated time points during 200 μM arsenite treatment; nuclei were labeled with DAPI; scale bar, 10 μm. (**B**) Quantification of SG area in wild-type and *CCHCR1*-KO cells at 45 and 60 min. SG area was quantified using ImageJ with automated particle analysis. At least 30 cells (*n* = 30) per condition were counted across three independent experiments. Data represent mean ± SD. **** *p* < 0.0001 (one-way ANOVA with Dunnett’s post hoc test). (**C**) Representative immunofluorescence images of wild-type (WT) and *CCHCR1*-KO (KO) cells during recovery from 400 μM arsenite. Cells were washed and allowed to recover in fresh medium for 60, 90, or 120 min. The cells were stained for G3BP1 (green) and DDX6 (red); nuclei were labeled with DAPI; scale bar, 20 μm. (**D**) Kinetics of SG disassembly following arsenite withdrawal. WT and *CCHCR1*-KO cells were treated with 400 µM arsenite for 40 min, washed, and recovered for the indicated times. The percentage of G3BP1-positive cells was quantified (*n* = 30 cells per condition, three independent experiments; mean ± SD). Significance was assessed by two-way ANOVA with Sidak’s post hoc test: ns, not significant; * *p* < 0.05; **** *p* < 0.0001.

To directly assess the role of CCHCR1 in SG assembly kinetics, we performed time-course analysis at 200 μM arsenite. This analysis revealed distinct UBAP2L redistribution kinetics between genotypes. In wild-type cells, UBAP2L appeared in G3BP1-positive SGs as early as 15 min and accumulated progressively through 60 min. In contrast, *CCHCR1*-KO cells showed delayed release of UBAP2L from P-bodies. At 15 and 30 min, UBAP2L remained predominantly DDX6-positive, with minimal SG entry. By 45 min, UBAP2L began appearing in SGs, and P-body signals correspondingly diminished. By 60 min, most UBAP2L had translocated to SGs in KO cells, though residual P-body signal persisted (Figure 4A). Quantification of SG area at 45 and 60 min showed ~71% and ~43% reduction in KO cells compared to wild-type, respectively (Figure 4B). Notably, SG number was comparable between genotypes at 60 min, with a slight tendency toward increased granule number in KO cells. These findings demonstrate that CCHCR1 deficiency delays UBAP2L incorporation into SGs.

We next examined SG disassembly kinetics upon stress withdrawal. Cells were treated with 200 μM arsenite for 60 min, washed, and allowed to recover in fresh medium for 60, 90, or 120 min. In wild-type cells, G3BP1-positive SGs persisted at 60 min post-withdrawal, with approximately 100% of cells retaining SGs. By 90 min, this proportion declined to 90%, and by 120 min, most SGs had resolved, though approximately 20% of cells retained a few persistent puncta. In contrast, CCHCR1-KO cells showed accelerated SG disassembly. At 60 min, 88% of cells contained G3BP1-positive SGs, and by 90 min, this dropped to 36%. By 120 min, SGs had completely disassembled in KO cells (Figure 4C,D). Together, these results suggest that CCHCR1 protects SGs from premature disassembly. Since DDX6 restricts SG assembly and promotes SG disassembly [9], these results strongly indicate that the aberrant DDX6-UBAP2L interaction in CCHCR1-deficient cells both impairs SG nucleation and accelerates SG clearance.

### 2.5. CCHCR1 Prevents UBAP2L-Driven P-Body–Stress Granule Fusion

Previous studies have shown that overexpression of UBAP2L induces hybrid granules containing both P-body and stress granule components, effectively merging SGs and PBs into single condensates. This phenomenon reflects the unique position of UBAP2L as a bridge between SG and P-body networks, mediated by its interactions with G3BP1/2 and DDX6. Notably, this hybrid granule phenotype is specific to UBAP2L among SG-nucleating proteins; overexpression of G3BP1 or FXR1 does not elicit equivalent fusion [14].

**Figure 5:**
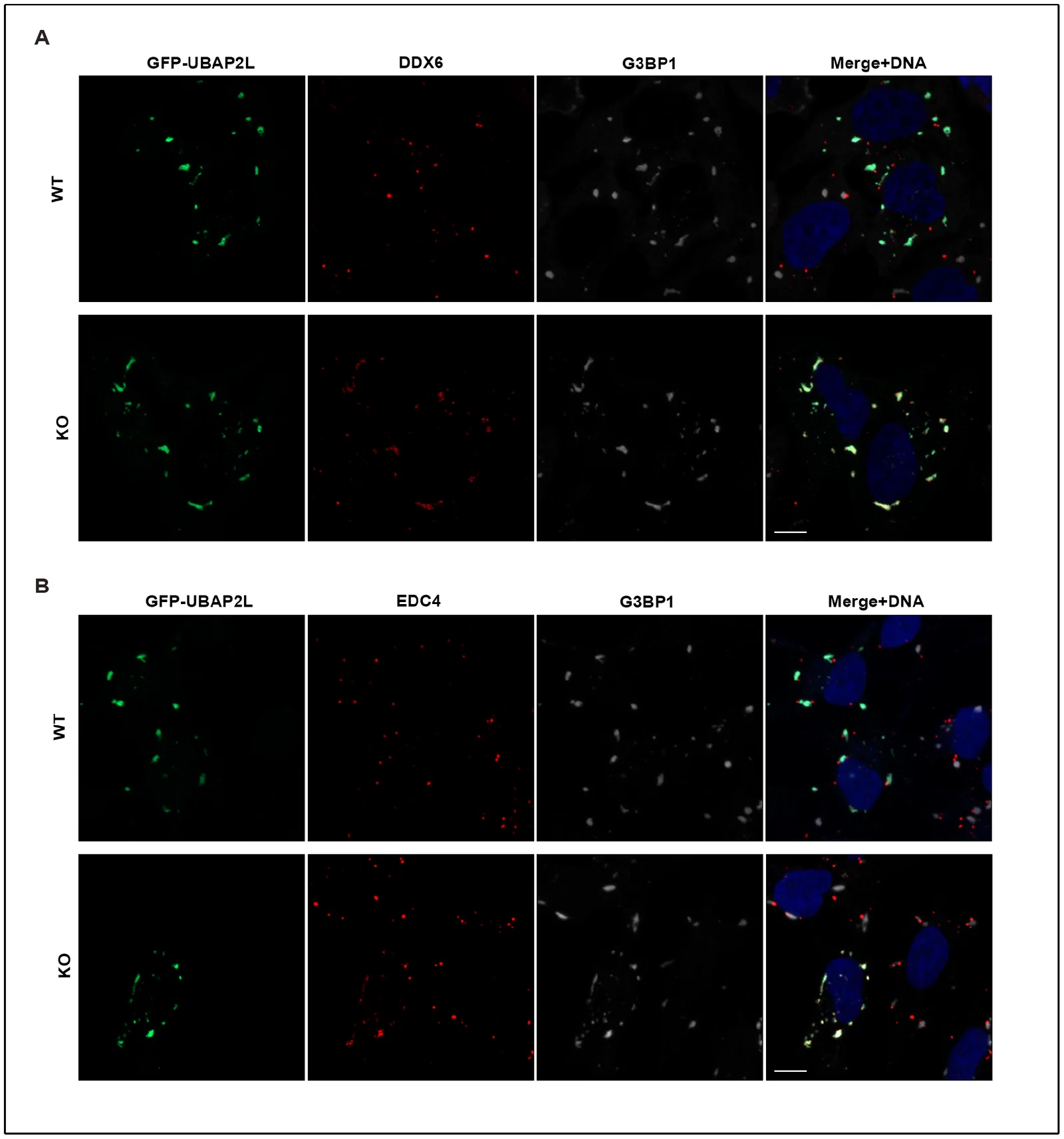
CCHCR1 prevents UBAP2L-driven P-body–stress granule fusion. (**A**) Wild-type (WT) or *CCHCR1*-KO (KO) HEK293 cells expressing moderate levels of GFP-UBAP2L (green), treated with 400 μM arsenite for 50 min, and stained for DDX6 (red) and G3BP1 (gray); nuclei were labeled with DAPI (blue); scale bar, 10 μm. (**B**) Wild-type or CCHCR1-KO HEK293 cells expressing moderate levels of GFP-UBAP2L (green), treated with 400 μM arsenite, and stained for EDC4 (red) and G3BP1 (gray). Nuclei were labeled with DAPI (blue); scale bar, 10 μm.

**Figure A6:**
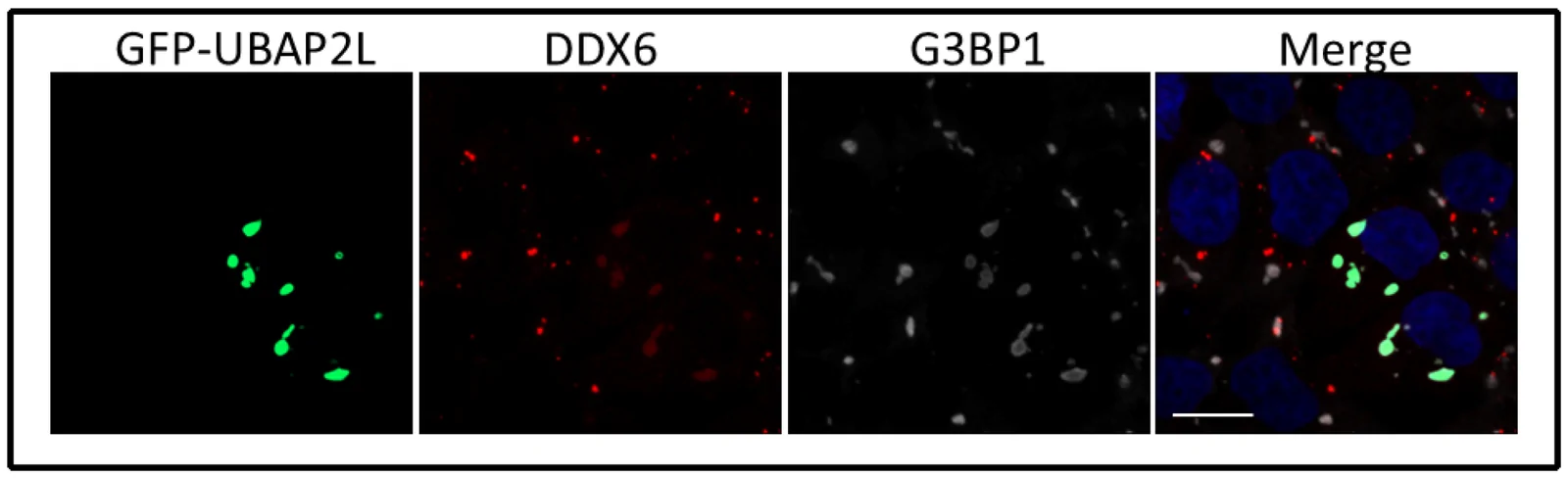
High-level overexpression of UBAP2L induces hybrid granules. HEK293 cells overexpressing GFP-UBAP2L (green) at high levels were treated with 400 μM arsenite and immunostained for G3BP1 (gray) and DDX6 (red). Note extensive co-localization of UBAP2L, G3BP1, and DDX6 within single condensates. Scale bar, 10 μm.

Next, we investigated whether CCHCR1 modulates this UBAP2L-driven hybridization. In HEK293 cells overexpressing GFP-UBAP2L at high levels and treated with 400 μM arsenite, we observed large hybrid granules positive for UBAP2L, G3BP1, and the P-body marker DDX6, with extensive signal overlap among all three components (Figure A6). However, when GFP-UBAP2L was expressed at moderate levels under the same conditions, UBAP2L and G3BP1 co-localized in large condensates in wild-type cells, while DDX6 signals appeared as smaller puncta that were largely non-overlapping and only partially adjacent to SGs. Strikingly, in CCHCR1-KO cells under identical conditions, DDX6 extensively co-localized with UBAP2L and G3BP1 in large hybrid condensates, with near-complete signal overlap, indicating loss of spatial segregation (Figure 5A). Identical results were obtained using another P-body marker, EDC4, confirming that the hybridization reflects complete P-body–SG fusion rather than DDX6-specific effects (Figure 5B).

### 2.6. CCHCR1 Rescues Stress Granule Assembly in Knockout Cells

To confirm that the defects in SG assembly observed in *CCHCR1*-KO cells were specifically due to loss of CCHCR1, we performed rescue experiments. *CCHCR1*-KO cells were transiently transfected with CCHCR1-GFP or GFP alone, treated with 100 μM arsenite for 50 min, and examined for SG formation using immunofluorescence staining for G3BP1.

**Figure 6:**
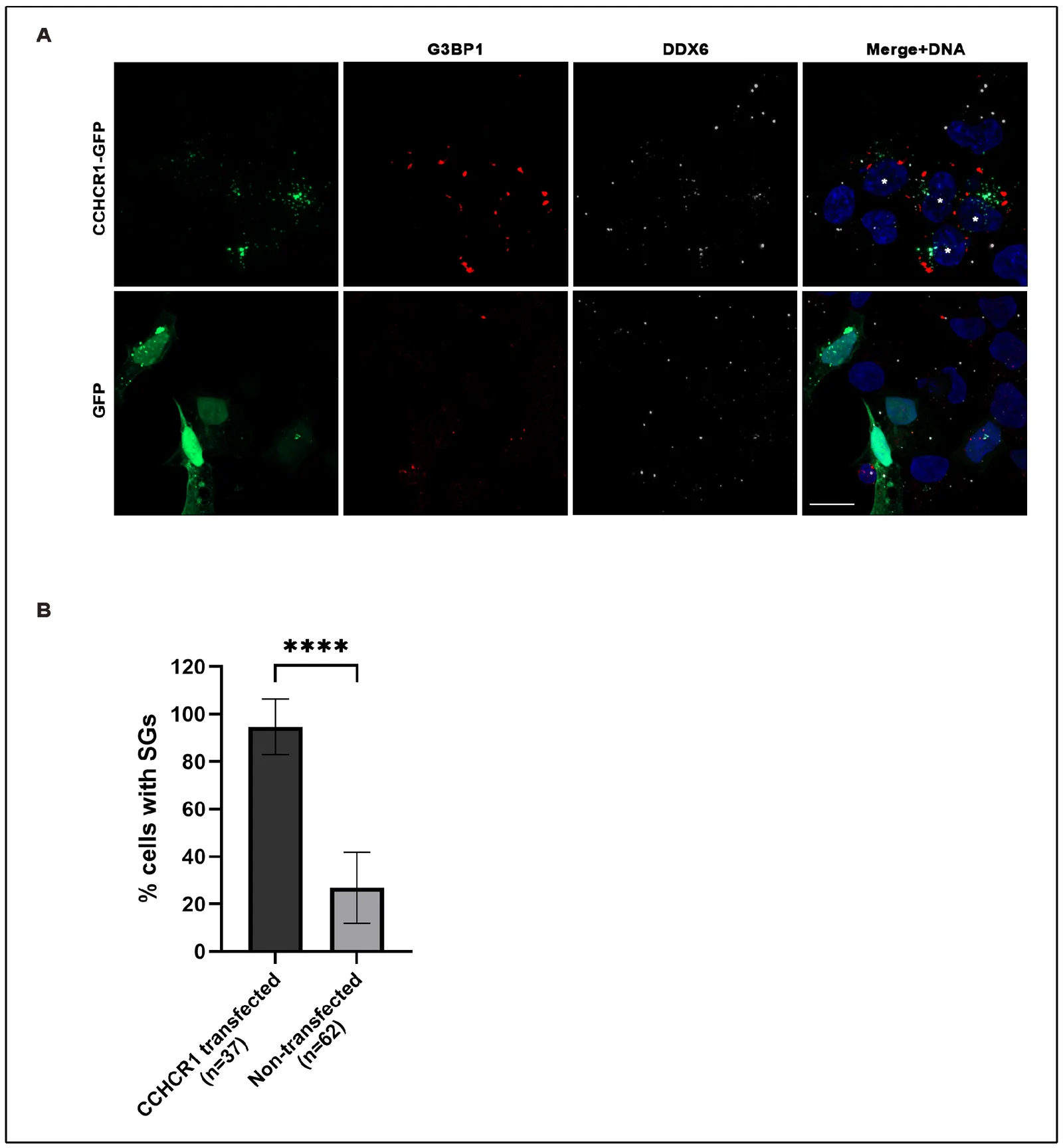
CCHCR1 rescues stress granule assembly in knockout cells. (**A**) Representative immunofluorescence images of *CCHCR1*-KO HEK293 cells transiently transfected with CCHCR1-GFP (upper panel) or GFP-only (lower panel), treated with 100 μM arsenite for 50 min, and that were stained for G3BP1 (red). Asterisks indicate CCHCR1-GFP positively transfected cells; nuclei were labeled with DAPI (blue); scale bar, 15 μm. (**B**) Quantification of SG formation in CCHCR1-GFP-transfected versus non-transfected KO cells. Data represent the mean ± SD. **** *p* < 0.0001 (Student’s *t*-test).

In cells transfected with CCHCR1-GFP, robust G3BP1-positive SGs were readily detected, whereas neighboring non-transfected cells within the same field of view showed no SG formation, with G3BP1 remaining diffuse in the cytoplasm (Figure 6A, upper panel). Quantification revealed that approximately 98% of CCHCR1-GFP-transfected KO cells formed SGs, compared to less than 25% of non-transfected cells (Figure 6B). In contrast, GFP-only transfected cells showed no SG formation, with diffuse G3BP1 staining indistinguishable from neighboring non-transfected KO cells (Figure 6A, lower panel). These results establish CCHCR1 as an essential regulator of SG assembly under stress.

## 3. Discussion

**Figure 7:**
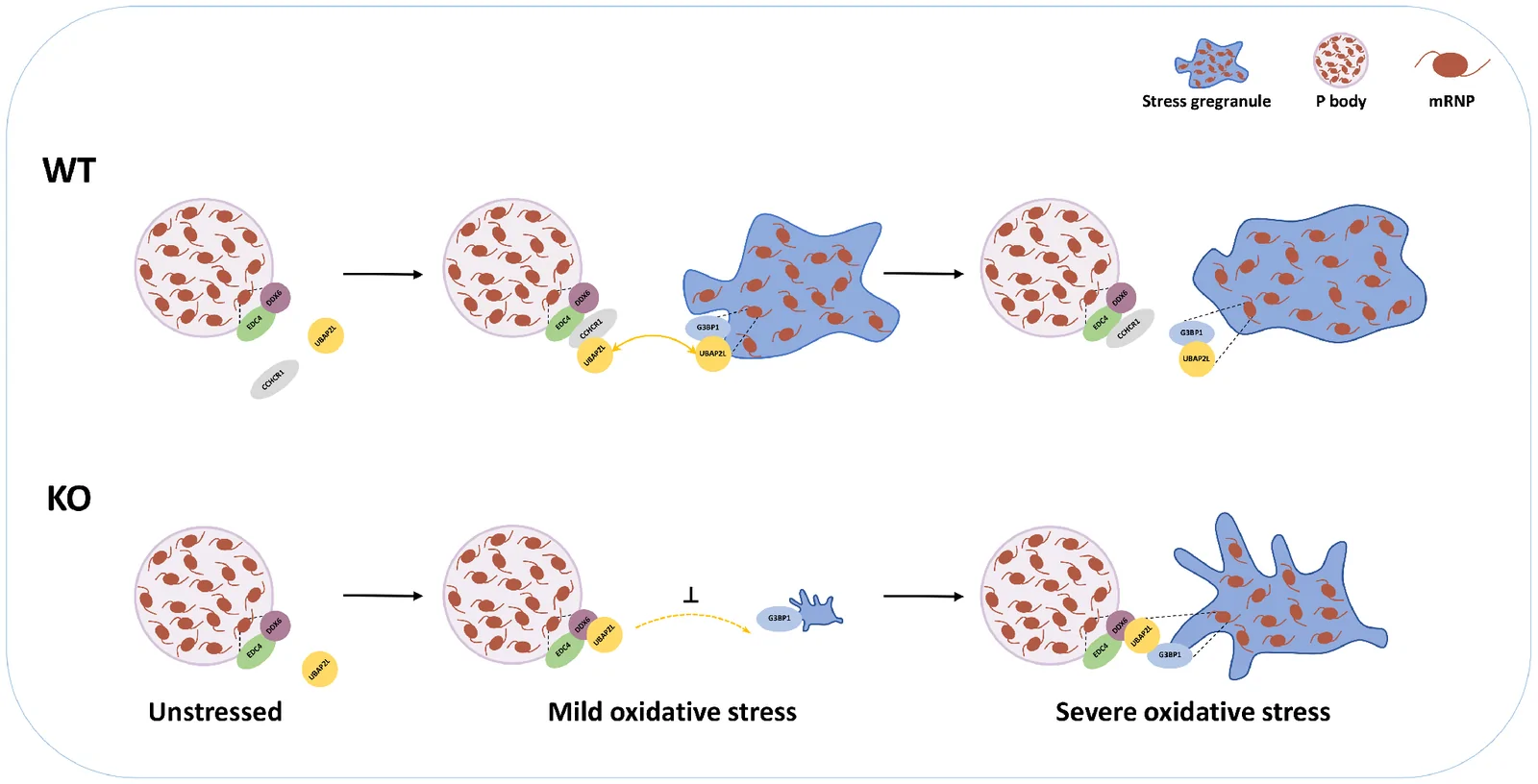
Model of CCHCR1-mediated UBAP2L partitioning between P-bodies and stress granules. In wild-type (WT) cells, both UBAP2L and CCHCR1 are diffusely distributed in the cytoplasm under basal conditions. Upon mild oxidative stress, CCHCR1 interacts with UBAP2L at P-bodies, where it prevents DDX6 from stably trapping UBAP2L in the P-body core. As stress intensifies, this interaction weakens, permitting UBAP2L release and subsequent incorporation into G3BP1-driven stress granules, thereby ensuring efficient SG assembly and P-body–SG spatial separation. In CCHCR1-deficient cells (KO), UBAP2L is aberrantly sequestered by DDX6 in P-bodies under mild-to-moderate stress, resulting in delayed SG assembly and reduced granule size. Under severe stress, residual UBAP2L eventually translocates to SGs, but spatial separation between the two compartments is compromised. Furthermore, elevated UBAP2L expression in the absence of CCHCR1 drives P-body–SG fusion, producing hybrid granules.

Although previous studies have characterized CCHCR1 as a P-body and centrosome-associated protein, our findings demonstrate that endogenous CCHCR1 is recruited to P-bodies specifically under oxidative stress. Through its interaction with UBAP2L, CCHCR1 restricts UBAP2L retention in P-bodies and thereby regulates its stress-dependent redistribution between P-bodies and stress granules. CCHCR1 deficiency causes aberrant UBAP2L accumulation in P-bodies, resulting in delayed SG assembly, reduced SG size, and increased SG–P-body proximity. We propose a model in which CCHCR1 functions as a competitive binding partner that regulates the hierarchical partitioning of UBAP2L between distinct membrane-less organelles. In mild oxidative stress conditions, CCHCR1 localizes to P-bodies through its CC1 domain and engages UBAP2L with moderate affinity. This interaction is functionally significant because it prevents UBAP2L from forming stable complexes with P-body core components such as DDX6. As stress intensifies, UBAP2L dissociates from CCHCR1 and is redirected to the G3BP1-driven stress granule nucleation machinery. The relatively weak affinity of the CCHCR1–UBAP2L interaction is thus advantageous: it permits stress-responsive release of UBAP2L from P-bodies, enabling efficient SG assembly without constitutive sequestration. In CCHCR1-deficient cells, UBAP2L is instead captured by DDX6 through a higher-affinity interaction, becoming trapped in P-bodies and only dissociating under more severe stress conditions to participate in SG assembly. The biochemical consequence is defective SG assembly: small, irregular granules with impaired maturation and disassembly kinetics (Figure 7). The stronger UBAP2L–DDX6 interaction also manifests morphologically: when UBAP2L is overexpressed in CCHCR1-KO cells, the DDX6-bound UBAP2L pool acts as a bridge that physically tethers P-bodies to forming stress granules, resulting in hybrid organelles. In wild-type cells, by contrast, moderate UBAP2L overexpression does not produce hybridization because the CCHCR1–UBAP2L interaction effectively buffers the DDX6-binding pool. Only when UBAP2L expression reaches supraphysiological levels that exceed the binding capacity of endogenous CCHCR1 does the DDX6-bound fraction become sufficient to drive P-body–SG fusion. This competitive binding model thus explains why CCHCR1 deficiency lowers the threshold for hybrid granule formation and why wild-type cells maintain robust SG-P-body compartmentalization across a broad range of UBAP2L expression levels.

UBAP2L is recruited to P-bodies not only under arsenite-induced oxidative stress but also under mild osmotic stress that induces P-bodies without assembling stress granules [14]. While constitutive P-bodies lack UBAP2L, stress-induced remodeling enables its entry, though the functional significance of this recruitment remains unclear. A recent study has established UBAP2L as a dynamic, bridge-forming component of the stress granule–P-body interaction network [14]. Our findings identify CCHCR1 as a regulatory layer that refines this model by revealing how UBAP2L’s bridging activity is restrained under physiological conditions. This mechanism explains why supraphysiological UBAP2L expression drives hybridization, while physiological levels maintain compartmentalization—a phenomenon previously documented but mechanistically unresolved. By identifying CCHCR1 as the competitive switch controlling this threshold, our work provides the mechanistic basis for UBAP2L-mediated organelle regulation.

DDX6 has been implicated in both restricting and promoting stress granule assembly. The ATPase and RNA-binding activities of DDX6 limit SG formation, and its depletion causes spontaneous granule enlargement [9]. DDX6 and the P-body scaffold GW182 also facilitate SG biogenesis by recruiting assembly factors to P-body platforms [29]. CCHCR1 may connect these functions through its interaction with UBAP2L. By binding UBAP2L within P-bodies, CCHCR1 could limit its interaction with DDX6 and preserve a pool for SG assembly. Alternatively, CCHCR1 may regulate UBAP2L distribution independently of DDX6. Distinguishing these possibilities will require further analysis of the CCHCR1–UBAP2L–DDX6 relationship. More broadly, the relative abundance of CCHCR1 and UBAP2L and the efficiency of CCHCR1 recruitment to P-bodies under stress are expected to determine the extent of UBAP2L retention by DDX6. Alterations in this balance would therefore shift the threshold for SG assembly and the strength of PB–SG contact, a prediction that warrants future investigation.

UBAP2L function is regulated by multiple post-translational modifications. Arginine methylation of the RGG motif by PRMT1 suppresses UBAP2L association with SG components and promotes SG disassembly [15]. O-GlcNAcylation enhances UBAP2L-driven SG formation and contributes to sunitinib resistance in renal cell carcinoma [16]. Whether CCHCR1–UBAP2L interaction is influenced by, or itself influences, the modification status of UBAP2L remains to be tested. Notably, CCHCR1 binding to the N-terminal region of UBAP2L encompasses the RGG motif and nearby residues that may carry O-GlcNAc modifications. It is therefore possible that CCHCR1 binding alters the accessibility of PRMT1 or OGT to UBAP2L or that pre-existing modifications on UBAP2L modulate CCHCR1 affinity. Such reciprocal regulation would provide an integrated mechanism for stress-responsive SG dynamics, and warrants further investigation.

Finally, whether CCHCR1 and UBAP2L are functionally linked in spindle regulation also deserves further investigation. UBAP2L has been reported to regulate PLK1 localization at kinetochores and its stability during mitotic exit [30]. We have previously shown that CCHCR1 interacts with Astrin, a spindle-associated protein required for kinetochore-microtubule attachment and centriole duplication. Given that both UBAP2L and Astrin participate in spindle assembly and chromosome segregation, the role of CCHCR1 in spindle regulation also deserves further investigation.

In summary, our findings establish CCHCR1 as a stress-responsive gatekeeper that translocates to P-bodies under arsenite exposure and regulates UBAP2L access to the PB core, thereby modulating both the availability of UBAP2L for stress granule assembly and the extent of PB–SG contact. The stress signals that direct CCHCR1 to P-bodies, and how they set the balance between DDX6-mediated UBAP2L retention and G3BP1-driven SG assembly, remain to be determined.

## 4. Materials and Methods

All antibodies and main reagents used in this study are listed in Supplementary Table S1.

### 4.1. Plasmids

CCHCR1-GFP and GFP-tagged truncation mutants (CC1–CC3) were constructed as previously described [24]. GST-CCHCR1-CC1 was generated by subcloning into the pGEX-4T-2 vector. GFP-UBAP2L was generated by PCR amplification of full-length human UBAP2L cDNA (NM_014847) from HEK293 cDNA and subcloning into the pEGFP-C3 vector (Clontech, Mountain View, CA, USA). GFP-UBAP2L truncation mutants (aa 1–528 and aa 529–1087) were similarly generated and subcloned into pEGFP-C3. UBAP2L (aa 1–528) was subcloned into pRSET-A (Invitrogen, Waltham, MA, USA) for bacterial expression of His-tagged protein. All constructs were verified by DNA sequencing.

### 4.2. Cell Culture

Human embryonic kidney 293 (HEK293) cells and Human osteosarcoma (U2OS) cells were sourced from the China Center for Type Culture Collection (CCTCC) and cultured in DMEM containing 10% FBS and 1% penicillin-streptomycin (100 U/mL and 100 μg/mL, respectively) at 37 °C with 5% CO_2_. The *CCHCR1*-knockout HEK293 cell line was generated via CRISPR-Cas9 and purchased from Ubigene Biosciences (Guangzhou, China). The following single guide RNAs (sgRNAs) were used for gene targeting: sgRNA-1 (5′-TTCCTGGGTCTTGCTAGGGTTGG-3′) and sgRNA-2 (5′-TGTGTGCAGACTTAGGGATCAGG-3′). Verification details are provided in Supplementary Figure S1.

### 4.3. Transfection

For plasmid transfection, cells were seeded into culture dishes 24 h in advance to achieve a confluence of 80–90% on the day of transfection. For each 35 mm culture dish, 2 μg of plasmid DNA and 4 μL of Lipofectamine 3000 (Invitrogen) were mixed according to the manufacturer’s instructions. The mixture was added to the cells, which were subsequently cultured for an additional 24–48 h. For siRNA transfection, cells were seeded into 35 mm culture dishes 24 h prior to transfection to reach 60–80% confluence. For each dish, 120 pmol siRNA was mixed with 6 μL Lipofectamine 3000 according to the manufacturer’s instructions and added to cells. After 24 h, cells were passaged at a 1:3 ratio and transfected again. Cells were harvested 24–48 h after the second transfection for subsequent analysis. The siRNA sequences of CCHCR1 have been previously reported [26].

### 4.4. Stress Induction

Cells were treated with sodium arsenite (AS) at indicated concentrations (50, 100, 200, or 400 μM) for 50 min, unless otherwise specified. For recovery experiments, cells were washed twice with PBS and cultured in fresh complete medium for the indicated times.

### 4.5. (Co)-Immunoprecipitation (IP)

For each reaction, 2 × 10^6^ cells were lysed with 1 mL of lysis buffer (50 mM Tris-HCl pH 7.5, 150 mM NaCl, 0.5% NP-40, and protease inhibitor cocktail) and incubated on ice for 30 min. Lysates were centrifuged at 16,000× *g* for 30 min at 4 °C. Then, 800 μL of supernatant was combined with 2 μg of the indicated antibody or 2 μg of nonspecific IgG of the same species as the control. The mixtures were rotated for 2 h at 4 °C, and then 30 μL of washed 50% protein A/G magnetic beads were added and continuously rotated at 4 °C overnight. After washing four times with lysis buffer, the beads were boiled in 40 μL of 2× sodium dodecyl sulfate (SDS) loading buffer for SDS–polyacrylamide gel electrophoresis (PAGE).

For endogenous co-immunoprecipitation across arsenite concentrations, cells were treated with indicated concentrations of sodium arsenite for 50 min prior to lysis.

### 4.6. GST Pull-Down Assay

His-UBAP2L (aa 1–528) was expressed in *E. coli* BL21(DE3) and purified using Ni-NTA agarose (Beyotime Biotechnology, Shanghai, China) according to the manufacturer’s instructions. GST-CCHCR1-CC1 and GST alone were transformed into E. coli BL21(DE3), and recombinant protein expression was induced at 18 °C with 0.1 mM IPTG. The bacteria were harvested, resuspended, sonicated, and centrifuged at 12,000 g for 30 min. Supernatants were incubated with glutathione magnetic beads for 2 h at 4 °C. After four washes with phosphate-buffered saline (PBS), the beads were incubated with purified His-UBAP2L (aa 1–528) for 4 h at 4 °C. Beads were then washed with PBS four times and boiled in 2× SDS loading buffer.

### 4.7. Western Blotting (WB)

Proteins were separated by SDS–PAGE and transferred to a nitrocellulose filter membrane. The membrane was blocked with blocking buffer (5% skim milk in Tris-buffered saline (TBS) containing 0.1% Tween 20) for 1 h and then probed with primary antibody diluted in the blocking buffer overnight at 4 °C. After washing four times with Tris-buffered saline with Tween 20 (TBS-T), the membrane was incubated with horseradish peroxidase-conjugated mouse or rabbit secondary antibody for 1 h at room temperature. After washing four times with TBS-T, the membrane was developed with enhanced chemiluminescent substrate and exposed using a chemiluminescent imaging system.

### 4.8. Immunofluorescence Microscopy

Cells grown on coverslips were fixed with 4% paraformaldehyde (PFA) for 15 min at room temperature and then permeabilized with 0.2% Triton X-100 for 10 min. After being blocked with blocking buffer, cells were sequentially incubated with primary and secondary antibodies coupled to Alexa Fluor 488, Alexa Fluor 555, or Alexa Fluor 647. To stain DNA, cells were incubated with 1 μg/mL 4′,6-diamidino-2-phenylindole (DAPI) for 10 min. Fluorescence images were collected with a Carl Zeiss LSM800 confocal laser scanning microscope (Carl Zeiss, Jena, Germany) and processed using ImageJ software (version 1.54) if necessary.

### 4.9. Quantification of P-Body-Associated UBAP2L Intensity

Immunofluorescence images were acquired using consistent acquisition settings across all experimental groups. P-body regions were segmented by thresholding the DDX6 channel with Otsu’s algorithm, generating binary masks that were subsequently overlaid onto the UBAP2L channel. Mean fluorescence intensity (MFI) of UBAP2L within masked P-body regions was extracted, with background correction performed by subtracting the average intensity of three cytoplasmic areas lacking P-body or SG signal. A minimum of 30 cells per condition were quantified across three independent experiments. Results are expressed as mean ± SD.

### 4.10. Quantification of Stress Granule Area

Following G3BP1 immunostaining, images were captured under uniform acquisition settings. Stress granule territories were delineated by applying Otsu’s auto-thresholding to the G3BP1 channel, yielding binary masks. Granule areas were computed by integrating pixel counts across all G3BP1-positive structures per cell, with particle size restricted to 0.5–5 μm^2^ to exclude noise and oversized aggregates. A minimum of 30 cells per condition were quantified across three independent experiments. Results are expressed as mean ± SD.

### 4.11. Statistical Analysis

Data distribution was assessed using the Shapiro–Wilk test. For comparison of two groups, Student’s *t*-test was used. For comparison of multiple groups, one-way analysis of variance (ANOVA) followed by Dunnett’s *t*-test for post hoc comparison against the control group was used. All statistical analyses were carried out in GraphPad Prism 8. Differences were considered statistically significant at *p* < 0.05.

## Data Availability

TIFF-format images and statistical analysis data supporting this study have been deposited in Zenodo and are openly available at https://doi.org/10.5281/zenodo.21057159. Raw CZI-format microscopy files are available from the corresponding author upon reasonable request.

## Supplementary Materials

The following supporting information can be downloaded at https://www.mdpi.com/article/10.3390/ijms27167451/s1.

## Abbreviations

The following abbreviations are used in this manuscript:

GST	Glutathione S-transferase
UBAP2L	Ubiquitin-binding associated protein 2-like
CCHCR1	Coiled-Coil Helicase Containing RNA-binding protein 1
CRISPR	Clustered Regularly Interspaced Short Palindromic Repeats
Cas9	CRISPR-associated protein 9
DDX6	DEAD-Domain-containing protein X 6
EDC4	Enhancer of Decapping 4
DCP1/2	Decapping Coprotein 1
XRN1	5′→3′ exoribonuclease 1
G3BP1/2	GTPase-activating protein SH3 domain-binding protein 1
PRMT1	Protein Arginine Methyltransferase 1
PSORS1	Psoriasis susceptibility locus 1
MCPH	Microcephaly Primary Hereditary
CEP72	Centrosomal Protein 72
WT	Wild Type
KO	Knock Out
SG	Stress Granule
P-body	Processing Body
AS	Arsenite (Sodium arsenite)
